# Respiratory syncytial virus seasonality in three epidemiological zones of Kenya

**DOI:** 10.1111/irv.12810

**Published:** 2020-12-10

**Authors:** Erica Billig Rose, Bryan O. Nyawanda, Patrick K. Munywoki, Nickson Murunga, Godfrey M. Bigogo, Nancy A. Otieno, Clayton Onyango, Sandra S. Chaves, Jennifer R. Verani, Gideon O. Emukule, Marc‐Alain Widdowson, D. James Nokes, Susan I. Gerber, Gayle E. Langley

**Affiliations:** ^1^ Division of Viral Diseases Centers for Disease Control and Prevention Atlanta GA USA; ^2^ Kenya Medical Research Institute ‐ Center for Global Health Research Kisumu Kenya; ^3^ Division of Global Health Protection Centers for Disease Control and Prevention Nairobi Kenya; ^4^ Kenya Medical Research Institute (KEMRI)‐Wellcome Trust Research Programme Kilifi Kenya; ^5^ Influenza Division Centers for Disease Control and Prevention Atlanta GA USA; ^6^ Influenza Program Centers for Disease Control and Prevention Nairobi Kenya; ^7^ Institute of Tropical Medicine Antwerp Belgium; ^8^ School of Life Sciences and Zeeman Institute (SBIDER) University of Warwick Coventry UK; ^9^ Division of Viral Diseases Centers for Disease Control and Prevention Atlanta GA USA

**Keywords:** respiratory syncytial virus, respiratory virus, RSV, RSV seasonality

## Abstract

Understanding respiratory syncytial virus (RSV) circulation patterns is necessary to guide the timing of limited‐duration interventions such as vaccines. We describe RSV circulation over multiple seasons in three distinct counties of Kenya during 2006‐2018. Kilifi and Siaya counties each had consistent but distinct RSV seasonality, lasting on average 18‐22 weeks. Based on data from available years, RSV did not have a clear pattern of circulation in Nairobi. This information can help guide the timing of vaccines and immunoprophylaxis products that are under development.

## 1 INTRODUCTION

Respiratory syncytial virus (RSV) is a leading cause of severe acute respiratory infections in children < 5 years of age worldwide, with most of the disease burden occurring in developing countries.^1^ RSV is estimated to cause 33.8 million cases of lower respiratory tract infections (LRTI) and at least 3.4 million hospitalizations globally.^1^ Studies have demonstrated a significant burden of RSV in Kenya with an annual RSV hospitalization rate estimated at 5 per 1000 among children < 5 years of age.^2^, ^3^, ^4^, ^5^, ^6^

Several RSV vaccines and immunoprophylaxis products targeting young infants are in development, and may be available within the next few years.^7^ However, the duration of protection of these products may be <6 months.^8^, ^9^ Therefore, understanding regional RSV circulation is necessary to guide the timing of clinical trials and administration of products once licensed.

Data on RSV circulation patterns in Kenya are sparse, and previous studies have been limited to one or two regions.^10^, ^11^ We describe RSV circulation over multiple seasons in three distinct counties of Kenya during 2006‐2018.

## 2 METHODS

**FIGURE 1:**
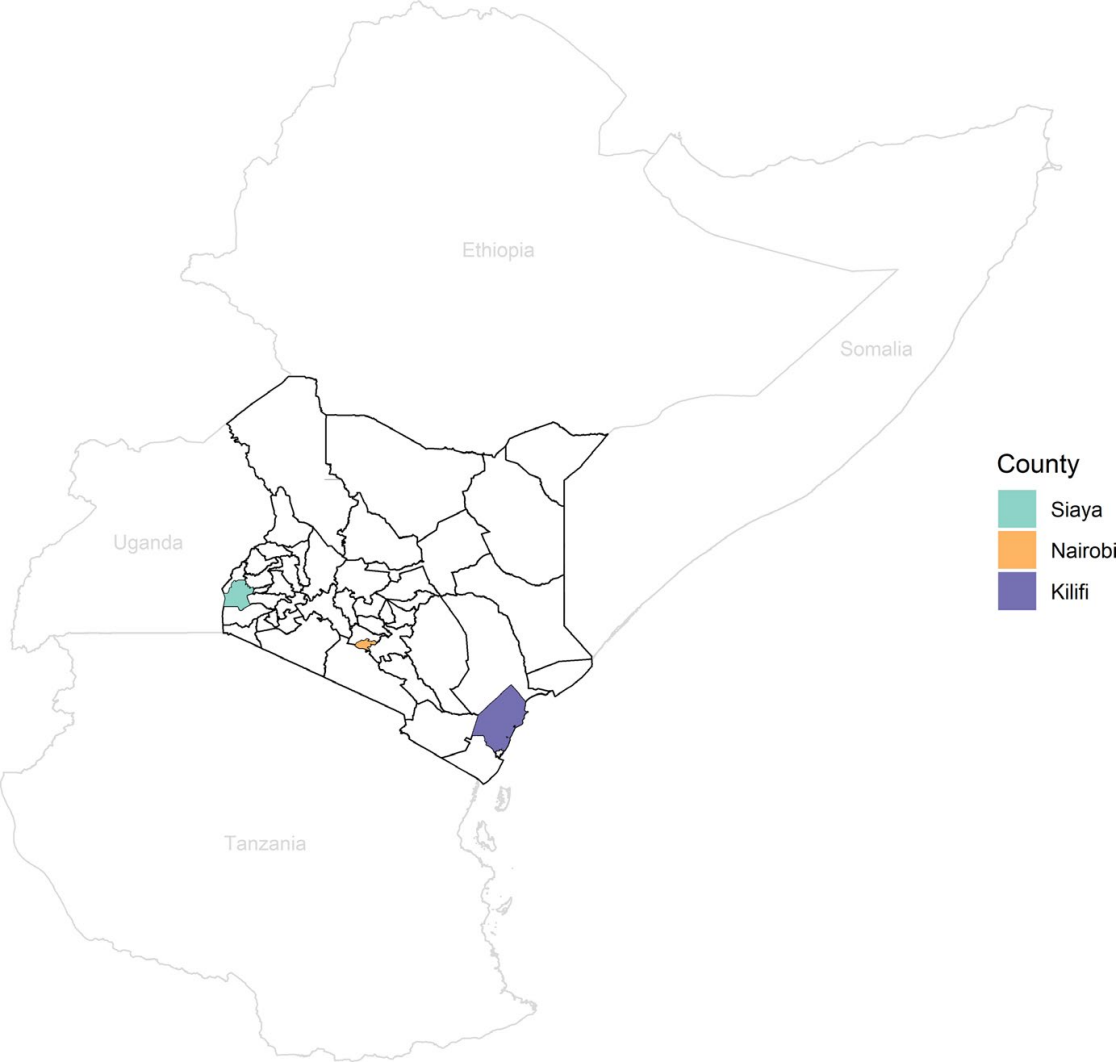
Map of Kenya and surrounding countries, including the three counties with RSV tests during the study period

All results of testing for RSV from the Kenya Medical Research Institute (KEMRI)‐Wellcome Trust Research Programme, and KEMRI‐Center for Global Health Research during the 13‐year period from 2006‐2018 were included in our analysis, regardless of laboratory testing methods, population tested, or the case definition used. Tested samples were collected from sites within three counties of Kenya: Kilifi, Nairobi, and Siaya (Figure 1).

In Kilifi, samples were collected from children aged 1 day to <5 years of age with syndromic severe or very severe pneumonia.^12^ In Nairobi, respiratory specimens were collected from all ages with influenza‐like illness (ILI) or acute lower respiratory illness (ALRI) at an outpatient clinic, Tabitha Clinic, and with severe acute respiratory illness (SARI) at an inpatient hospital, Kenyatta National Hospital. In Siaya, respiratory samples were collected from 3 inpatient hospitals and 3 outpatient clinics from patients of all ages with varying case definitions, including ILI, ALRI, SARI, and acute respiratory illness (ARI). See Table 1 for details of site testing populations, case definitions, and dates of specimen collection.

**Table 1:** Summary of specimens tested for RSV by region

Region	Location	Population tested	Study period	Testing method	Number tested	Number RSV positive (%)
Kilifi^a^	Kilifi County Hospital	Syndromic severe or very severe pneumonia^b^, 1 d to <5 y	1/06‐12/18	Antigen	9572	2477 (26)
			1/07‐10/18	Molecular		
Nairobi	Kenyatta National Hospital	SARI^c^, all ages	8/14‐3/16	Molecular	867	155 (18)
	Tabitha Clinic	ILI^d^ or ALRI^e^, all ages	10/06‐4/11	Molecular	4707	532 (11)
Siaya	Bondo Sub‐County Hospital and Siaya County Referral Hospital	ARI, pregnant women^f^ and infants^g^	1/15‐11/18	Molecular	3738	185 (5)
	Lwak Mission Hospital, Abidha Clinic, Madiany Sub‐county Referral Hospital, Mahaya Clinic, Ong'ielo Clinic, Siaya County Referral Hospital	ILI^d^ or ALRI^e^, all ages	1/06‐11/12	Molecular	4890	542 (11)
	Siaya County Referral Hospital	SARI^c^, all ages	8/09‐11/13	Molecular	5654	550 (10)
	Siaya County Referral Hospital	SARI^h^, all ages	12/13‐11/18	Molecular	2294	130 (6)
Total					31 722	4571 (14)

^a^ Some specimens tested by antigen and multiple molecular methods.

^b^ Syndromic severe or very severe pneumonia: cough or difficulty in breathing for <30 d, with lower chest wall indrawing, prostration (including inability to feed or drink), coma, or hypoxemia (pO_2_ < 90%).

^c^ Severe acute respiratory infection (SARI): cough and reported fever or a recorded temperature of ≥38°C, with an onset within the last 14 d.

^d^ Influenza‐like illness (ILI): cough or sore throat with axillary temperature ≥ 38°C with an onset within the last 14 d.

^e^ Acute lower respiratory infection (ALRI): for ≥5 y of age: cough or difficulty breathing or chest pain, with axillary temperature ≥ 38.0°C, oxygen saturation level ≤ 90% and/or hospitalization, with onset within the last 14 d; For <5 y of age: cough or difficulty breathing, and at least one of indrawing, oxygen saturation level ≤90%, stridor, inability to feed or drink, vomiting everything, lethargy, unconscious, convulsions, or hospitalization.

^f^ Acute respiratory infection, pregnant women (ARI): cough or difficulty breathing within the past 10 d.

^g^ ARI, infants: cough, difficulty breathing, runny nose or other respiratory illness within the past 10 d.

^h^ SARI: cough and reported fever or a recorded temperature of ≥38°C, with an onset within the last 10 d.

We used the number of specimens tested and the number of RSV detections to describe the percent positive by week within each region. To describe the season, we took the 5‐week moving average (previous 2 weeks, current week, and two subsequent weeks) of the weekly percent positive in each county, similar to previous approaches.^11^, ^13^ We described whether a consistent season was observed, and if so, the onset, offset, and peak weeks within each county. We defined season onset as the first of three consecutive weeks during which the moving average percentage positive was greater than the mean of the 5‐week moving average percentage positive for that calendar year. We defined the season offset as the third of three consecutive weeks following season onset by at least 5 weeks during which the moving average percentage positive was below the average of the 5‐week moving average percentage positive. The peak of the season was defined as the highest moving average percentage positive between the season onset and offset. We define the sensitivity of the season as the percentage of detections that occurred during each defined season, inclusive of the onset and offset.

## 3 RESULTS

Overall between January 1, 2006, and December 31, 2018, 31 722 RSV tests were collected from Kilifi, Nairobi, and Siaya counties. In Nairobi and Siaya, all tests were done by molecular methods. In Kilifi, either antigen or molecular methods were used. Overall, 4571 (14%) tests were positive for RSV (Table 1).

In Kilifi, all 9572 tests were collected at Kilifi County Hospital, of which 2477 (26%) were positive for RSV. In Nairobi, 5574 tests were collected, of which 687 (12%) were positive for RSV. In Siaya, 16 576 tests were collected from six surveillance sites, of which 1407 (8%) were positive for RSV (Table 1).

**FIGURE 2:**
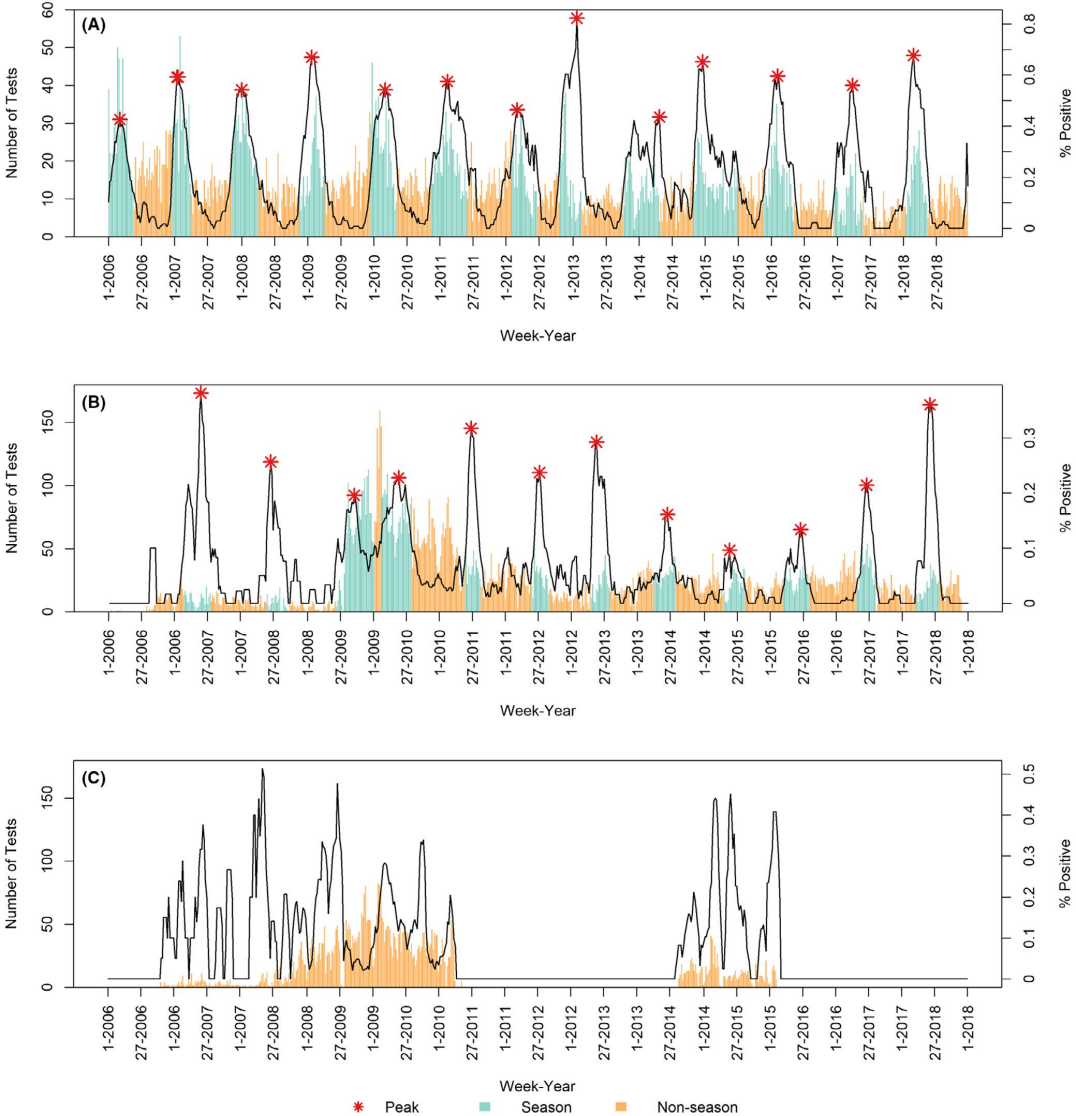
The number of specimens that were tested for RSV (bars) and the 5‐mo moving average percentage that were positive (black line) by week and county. In regions with a distinct season (Kilifi and Siaya), each season is shown in blue (inclusive of onset and offset), and each season peak is indicated by a red star. A. Kilifi, B. Siaya, C. Nairobi

Kilifi and Siaya each had consistent but distinct seasonality (Figure 2). Testing for RSV was discontinued in Nairobi from May 2011 until August 2014. Based on available years, RSV did not have a clear pattern of circulation in Nairobi, and we could not define season onset, offset, or peak for that region (Figure 2, Table 1).

The season onset in Kilifi occurred between mid‐October and early February each year, and season offset occurred between late March and mid‐June. The average season duration was 22 weeks (range: 16‐29 weeks). The peak occurred between late December and late April, and most commonly occurred in February. The defined season captured 81%‐97% of detections each year, and across all years, the median sensitivity of the season was 88% (Table 2).

**Table 2:** Season onset, offset, and peak for Kilifi and Siaya counties

Years	Kilifi				Siaya			
	Onset (Week)	Peak (Week)	Offset (Week)	Sensitivity (%)	Onset (Week)	Peak (Week)	Offset (Week)	Sensitivity (%)
2005‐2006	‐	March 5, 2006 (10)	May 14, 2006 (20)	89	‐	‐	‐	‐
2006‐2007	December 17, 2006 (51)	January 14, 2007^a^ January 21 2007^a^ (3‐4)	March 25, 2007 (13)	90	February 25, 2007 (9)	May 27, 2007 (22)	July 15, 2007 (29)	82
2007‐2008	November 11, 2007 (46)	December 30, 2007 (1)	April 6, 2008 (14)	87	May 25, 2008 (22)	June 15, 2008 (25)	September 7, 2008 (37)	81
2008‐2009	November 23, 2008 (47)	January 25, 2009 (4)	April 5, 2009 (14)	88	June 14, 2009 (24)	September 20, 2009 (38)	January 3, 2010 (1)	99
2009‐2010	December 20, 2009 (51)	March 7, 2010 (10)	May 9, 2010 (19)	90	February 21, 2010 (8)	May 23, 2010 (21)	July 25, 2010 (30)	69
2010‐2011	November 21, 2010 (47)	February 13, 2011 (7)	May 29, 2011 (22)	86	May 29, 2011 (22)	June 26, 2011 (26)	August 14, 2011 (33)	63
2011‐2012	February 5, 2012 (6)	March 4, 2012 (10)	June 17, 2012 (25)	86	May 27, 2012 (22)	July 8, 2012 (28)	August 26, 2012 (35)	63
2012‐2013	October 28, 2012 (44)	January 27, 2013 (4)	February 24, 2013 (8)	81	April 21, 2013 (17)	May 19, 2013 (31)	July 28, 2013 (21)	79
2013‐2014	October 20, 2013 (42)	April 27, 2014 (17)	May 4, 2014 (18)	85	April 6, 2014 (15)	June 15, 2014 (25)	July 27, 2014 (31)	65
2014‐2015	November 9, 2014 (45)	December 21, 2014 (51)	July 5, 2015 (27)	88	April 26, 2015 (17)	May 24, 2015 (21)	August 16, 2015 (33)	77
2015‐2016	November 20, 2015 (48)	February 7, 2016 (6)	May 8, 2016 (19)	97	March 20, 2016 (12)	June 19, 2016 (25)	August 7, 2016 (32)	96
2016‐2017	December 25, 2016 (52)	March 26, 2017 (13)	May 21, 2017 (21)	86	April 30, 2017 (15)	June 18, 2017 (25)	August 6, 2017 (32)	92
2017‐2018	January 21, 2018 (4)	February 25, 2018 (9)	May 13, 2018 (20)	93	March 25, 2018 (13)	June 3, 2018 (23)	July 22, 2018 (30)	98

^a^ Tied for peak percentage positive.

Siaya season onset occurred between late February and mid‐June each year, and season offset occurred between mid‐July and early September, except for the 2009 season which had an atypical offset in January. We could not adequately describe the 2006 season in Siaya due to low number of tests (N = 168). The average season duration was 18 weeks (range: 14‐23 weeks). The peak occurred between mid‐May and early July, except the 2009 season during which the peak occurred in September. Most commonly, the peak of the season occurred in June. The defined season captured 63%‐99% of detections each year, and across all years, the median sensitivity of the season was 80%.

## 4 DISCUSSION

The observed circulation patterns of RSV differed between regions of Kenya. In the two regions where RSV season could be defined, there was little overlap, with the peak generally occurring in Kilifi (Eastern coastal Kenya) between late December and late April and in Siaya (Western Kenya) between mid‐May and early July. With available data, the season could not be defined in the Nairobi region. These data may be used to make evidence‐based decisions in Kenya about optimal timing of vaccine and immunoprophylaxis products that may be licensed in the near future, as these products will provide limited duration of protection. Appropriately timing implementation of products with limited duration could be challenging in regions with varying and inconsistent patterns of circulation.

Our findings in Kilifi are consistent with previous studies, which also found the peak occurred on average in February during 2002‐2006.^10^, ^12^ Previous studies found distinct, but inconsistent seasons in Nairobi,^10^, ^11^, ^14^ but these analyses, like ours, were limited to only a few years. It is possible that additional data over longer continuous periods of time would reveal a specific RSV seasonality in the region. Previous studies did not find distinct seasons in Siaya during 2007‐2010, but our analysis over a longer period of time generally showed a distinct and consistent season in this county, with the exception of 2009.^11^, ^15^ The atypical findings from that year may have been the result of the influenza A/H1N1pdm09 pandemic's effect on testing and circulation of other viruses.^3^, ^11^

In tropical climates, some studies showed that RSV circulation patterns may be less defined, although other studies found tropical regions to have predictable circulation patterns.^10^ Despite both having tropical climates and rural settings, two of the three counties studied had consistent RSV seasons that were distinct from each other. There was wide variation in season onset and offset making seasons somewhat variable, but still a distinct season can be recognized. In Kilifi, >80% of detections each year were captured by the defined season. In Siaya, >62% of detections each year and >90% of detections in the last 3 years of this analysis were captured each season. In addition to understanding the duration of the season, recognizing peaks in infections is important to maximize the number of cases prevented when interventions are of limited duration. Several additional factors may influence the observed RSV seasonality, including access to healthcare, social and behavior characteristics, and susceptibility to infection.

Our study has several limitations. First, we compiled all available laboratory‐confirmed RSV tests in the three regions of our study, but testing practices, case definitions, age ranges, and methods varied by site and year. These differences may impact the sensitivity and specificity of RSV detections and the season duration. However, we believe that by comparing the percentage of positive tests relative to other points in time, we likely captured overall circulation patterns. This approach has also been used in previous studies.^13^ Second, data from Nairobi were limited, and with more data, a distinctive pattern may emerge. Third, we did not include any demographic information or information on clinical severity. We can only assume that the observed patterns held true across age groups and that interventions covering the defined seasons would prevent the most severe cases.

Understanding RSV seasonality is important to provide recommendations for optimal timing of interventions against RSV infection, particularly ones that may offer protection for a few months. This is particularly true for low‐ and middle‐income countries where competing priorities make optimizing resources essential. More studies on a regional level in low‐ and middle‐income countries are important to understand RSV patterns of circulation prior the licensure of products that are currently in development.

## CONFLICT OF INTEREST

I declare that there is no conflict of interest.

## AUTHOR CONTRIBUTIONS

**Erica Billig Rose:** Formal analysis (equal); Methodology (equal); Visualization (equal); Writing‐original draft (equal); Writing‐review & editing (equal). **Bryan O Nyawanda:** Conceptualization (equal); Data curation (equal); Writing‐review & editing (equal). **Patrick K. Munywoki:** Data curation (equal); Writing‐review & editing (equal). **Nickson Murunga:** Data curation (equal); Writing‐review & editing (equal). **Godfrey M. Bigogo:** Data curation (equal); Writing‐review & editing (equal). **Nancy A Otieno:** Data curation (equal); Writing‐review & editing (equal). **Clayton Onyango:** Data curation (equal); Writing‐review & editing (equal). **Sandra Chaves:** Conceptualization (equal); Supervision (equal); Writing‐review & editing (equal). **Jennifer R Verani:** Conceptualization (equal); Data curation (equal); Writing‐review & editing (equal). **Gideon Emukule:** Data curation (equal); Writing‐review & editing (equal). **Marc‐Alain Widdowson:** Supervision (equal); Writing‐review & editing (equal). **David James Nokes:** Conceptualization (equal); Supervision (equal); Writing‐review & editing (equal). **Susan I. Gerber:** Conceptualization (equal); Supervision (equal); Writing‐review & editing (equal). **Gayle Fischer:** Conceptualization (equal); Supervision (equal); Writing‐review & editing (equal).

### PEER REVIEW

The peer review history for this article is available at https://publons.com/publon/10.1111/irv.12810.
